# Divergence in the sow vaginal microbiota is associated with fertility

**DOI:** 10.1530/REP-25-0044

**Published:** 2025-06-18

**Authors:** Lauren Fletcher, Xiaoshu Zhan, Yashu Song, Julang Li

**Affiliations:** ^1^ Department of Animal Biosciences, University of Guelph, Guelph, Ontario, Canada; ^2^ School of Animal Science and Technology, Foshan University, Foshan, Guangdong, China

**Keywords:** fertility, vaginal microbiota, breeding herd, swine, machine learning

## Abstract

**In brief:**

Vaginal microbiota composition influences female fertility, however it has not been studied for measuring fertility level in female pigs. This study reveals significant vaginal microbiota composition differences between high reproductive performance and infertile sows, and demonstrates that the vaginal microbiota has promise for improving female pig selection using machine learning modeling.

**Abstract:**

There is a need for reliable and effective biomarkers of female fertility and reproductive potential in the pork industry, as current selection protocols are not keeping up with the rate of improvement for other production-related traits. This study aimed to investigate the vaginal microbiota composition between sows of differing fertility status and identify candidate vaginal microbiota biomarkers of sow fertility. The vaginal microbiota of high reproductive performance sows (HRP, *n* = 52) with number of piglets born alive ≥13 and infertile sows (INF, *n* = 23), that remained nonpregnant after two consecutive rounds of artificial insemination, were investigated. Sequencing results revealed significantly different (*P* < 0.05) beta diversity at the genus level between HRP and INF vaginal microbiota communities. Accordingly, the composition of the vaginal microbiota diverged between HRP and INF sows, with INF sows having increased (*P* < 0.05) relative abundance of *Lachnospiraceae XPB1014 group* and HRP sows having increased (*P* < 0.05) relative abundance of *Aerococcus* and *Staphylococcus* at the genus level. Forty-two genera were selected as candidate biomarkers of sow fertility via partial least squares discriminant analysis (PLS-DA) and recursive feature elimination. The support-vector machine model classified sow fertility with 93.3% accuracy, supporting potential industry application to improve upon current methods for selection and recruitment in the breeding herd. Future investigations should validate the candidate vaginal microbiota biomarkers in a large, independent population of sows and gilts to evaluate their application for predicting future reproductive performance and assess their true industry applicability.

## Introduction

The selection of female pigs for fertility and reproductive success is essential to promote sow lifetime productivity and retention in the breeding herd (Patterson & Foxcroft 2019). Genetics (Lillehammer *et al.* 2011, Knol *et al.* 2016) and metabolomics (Fletcher *et al.* 2022) are among the factors that can influence or reflect a female pig’s fertility and reproductive potential and have been explored for their application to commercial breeding herd selection. However, the composition of the pig vaginal microbiota has not been investigated in the context of female pig fertility status or for its capacity to aid breeding herd selection protocols through its association with female pig fertility or reproductive potential level. Given that the vaginal microbiota resides in the female reproductive tract, it likely plays a role in female pig conception and pregnancy success. In addition, due to its accessibility, it may be a convenient and valuable source of candidate biomarkers of fertility status.

The vaginal microbiome is a dynamic and interactive system with a mutualistic goal of host-microbiome homeostasis (Chen *et al.* 2021). Microbes that colonize the vagina associate with the vaginal epithelium for resources and secrete antimicrobial factors in return, such as bacteriocin, hydrogen peroxide or lactic acid, that help to maintain the health of the reproductive tract and to prevent the growth of pathogenic bacteria through competitive exclusion or killing (Kaewsrichan *et al.* 2006, Ghartey *et al.* 2014). The composition of the microbiota can be influenced by internal factors, including genetics, age, stage in the menstrual cycle and pregnancy, and external factors, such as diet, antibiotics, hygiene and microenvironment (Ravel *et al.* 2011). These factors can alter vaginal microbiome homeostasis, resulting in the loss of beneficial microbes, overgrowth of possibly harmful microbes and change in diversity. Accordingly, a wide range of negative reproductive states have been linked to shifts in human vaginal microbiota composition, including decreased IVF conception rates, increased risk of incidence of sexually transmitted infections, implantation failure, pregnancy loss, preterm birth and infertility (Fettweis *et al.* 2019, Haahr *et al.* 2019, Juliana *et al.* 2020, Grewal *et al.* 2022, Patel *et al.* 2022). Thus, the composition of the vaginal microbiota appears to be associated with fertility and reproductive health, suggesting that it may be an effective source of candidate biomarkers for fertility.

The literature on the female pig reproductive tract microbiome has been previously reviewed (Monteiro *et al.* 2022), highlighting investigations of urogenital infection (Wang *et al.* 2017, Zhang *et al.* 2021*b*), low and high reproductive performance after porcine reproductive and respiratory syndrome (PRRS) vaccination (Sanglard *et al.* 2020), and risk of pelvic organ prolapse in late gestation (Kiefer *et al.* 2021). Additional studies have investigated the vaginal microbiota between healthy sows and those with purulent discharge (Poor *et al.* 2022), sows with normal return-to-estrus and those without (Zhang *et al.* 2021*a*) and nonpregnant gilts and pregnant sows (Luque *et al.* 2021). However, vaginal microbiota composition relating to the fertility status of gilts or sows, specifically high reproductive performance and infertility, has not been investigated. Here, the composition of the vaginal microbiota of high reproductive performance (HRP) and infertile (INF) sows was investigated using 16S sequencing along with beta diversity, alpha diversity and relative abundance analysis. In addition, this study attempted to identify candidate vaginal microbiota biomarkers of sow fertility using a machine learning modeling approach.

## Materials and methods

### Animals and housing

All animal procedures were carried out in accordance with the Canadian Council on Animal Care (CCAC) guidelines under the Animal Utilization Protocol (AUP) #4825 approved by Animal Care Services (ACS) at the University of Guelph. Sows were either Yorkshire or Yorkshire-Landrace crosses that were individually housed in stalls or a group setting with access to water and restricted feed at Arkell Research Station (University of Guelph, Guelph, ON) or Alliance Genetics Canada’s affiliated operations. All animals included in the study were from 1 to 3 years of age, healthy, with no signs of illness or disease, and without abnormal vaginal discharge. HRP sows were between parity 1 and 6 with ≥13 piglets born alive on average throughout their reproductive lifespan immediately before sampling. Infertile sows (INF) were between parity 1 to 4 that remained nonpregnant after two rounds of back-to-back artificial insemination immediately before sampling (consecutive estrous cycles). All artificial inseminations were performed by experienced technicians using commercially sourced semen quality tested for sperm concentration, viability, motility, morphology and fertilization ability.

### Sample collection

Vaginal microbiome samples were taken in triplicate from 77 sows (HRP: *n* = 53, INF: *n* = 24) fitting the above assessment criteria. HRP sows were sampled 4 to 5 days after weaning, an approximate time of commencement of estrus in sows (Soede *et al.* 2011). INF sows were sampled during their next expected estrous cycle after remaining nonpregnant for two heat cycles. Sampling occurred 21–23 days after their last detected estrus, or, 3 to 5 days post-injection if P.G. 600^®^ was used for estrus synchronization. Estrus checks were performed following standard management practices using the standing response to back pressure test and visual changes in the vulva. All sows were indicated to be receptive for breeding by management when sampled. This was done to limit variability based on stage in the estrous cycle (Soede *et al.* 2011). Samples were also collected in the morning within the same 2-hour time window to limit variation based on circadian and diurnal variation. The outer vulva was washed with water and sprayed with 70% ethanol to ensure a pure vaginal microbiota sample collection. Swabs were inserted approximately 10–15 centimeters into the vagina, gently rotated for 20 s and immediately placed into PBS buffer on ice for transport, ensuring that the swab or vessel did not contact other surfaces. Upon arrival to the lab, the swabs were centrifuged at 9,000 ×***g*** for 5 min to extract the bacterial biomass suspended in the swab. The recovered biomass was resuspended and added back into the PBS and bacteria solution, creating a homogenous sample suspension. A combined sample, an equal ratio of each replicate sample, was pooled and stored at −80°C until further analysis.

### DNA extraction, 16S rDNA amplification and PacBio sequencing

Microbiome DNA extraction was performed using the Norgen Microbiome DNA Isolation Kit (Norgen Biotek, Canada) following manufacturer directions. Final extracted DNA was eluted with nuclease-free water. DNA was concentrated using the Thermo Scientific Savant™ SpeedVac™ DNA 130 Integrated Vacuum Concentrator System (Thermo Fisher Scientific, Canada) at ambient temperature to obtain a final volume of 20 μL.

The extracted microbiome DNA was amplified and prepared for 16S rRNA PacBio sequencing in a two-step PCR method. PCR1 was performed with primers Bact-27F (/5AmMC6/GCAGTCGAACATGTAGCTGACTCAGGTCACAGRGTTYGATYMTGGCTCAG) and Univ-1492R (/5AmMC6/TGGATCACTTGTGCAAGCATCACATCGTAGRGYTACCTTGTTACGACTT) (Frank *et al.* 2008), with universal PacBio adapters and relevant amino blocker modifications on the 5′ end to prevent ligation of the PCR product during SMRTbell library prep. The PCR1 primer sets were synthesized by Integrated DNA Technologies (IDT, USA). Each PCR1 reaction included 2 μL of the concentrated vaginal microbiome DNA, 2 μL 10× PCR buffer, 2 μL 50 mM MgCl_2_, 1.5 μL 2 mM dNTPs, 0.5 μL of 5 U/μL Taq Polymerase (Invitrogen™, USA), 1 μL 10 μM 27F primer, 1 μL 10 μM 1492R primer and 10 μL of molecular grade water for a final reaction volume of 20 μL. Samples were run on the CFX Connect Real-Time PCR Detection System Thermocycler (BioRad^®^, USA) under the following conditions: 94°C for 5 min, followed by 20 cycles of 94°C for 30 s, 65°C for 30 s and 72°C for 2 min and a final extension step at 72°C for 10 min. PCR1 products were stored at −20°C until further preparation. Test aliquots of random samples were visualized on 1% (*w*/*v*) agarose gel in SB buffer to ensure sufficient amplification before moving to the next step of the protocol.

PCR2, library preparation and sequencing were performed at the Centre for Biodiversity Genomics at the University of Guelph (Guelph, ON, Canada). PCR2 used PacBio unique molecular identifier (UMI) primers. The PCR2 cocktail for long PCR1 products per reaction was as follows: 13 μL 10% Trehalose, 2.8 μL 10× PCR Buffer, 1.3 μL 50 mM MgCl_2_, 1.3 μL 100% DMSO, 1.3 μL 0.2 mg/mL BSA, 0.5 μL of 10 μL of each UMI Primer (forward and reverse), 0.4 μL 10 mM dNTPs, 0.3 μL 5 U/μL Platinum Taq Polymerase (Invitrogen™) and 4.2 μL 10× diluted PCR1 DNA. The primers were added using Beckman Coulter’s Biomek FX^P^ Liquid Handling Automation System. Samples were run on a thermocycler under the following conditions: 94°C for 2 min, 25 cycles at 94°C for 40 s, 64°C for 1 min, 72°C for 2 min and 72°C for 10 min. PCR2 products were quality checked on a 2% (*w*/*v*) agarose gel. Each reaction was pooled into the DNA library by adding 6 μL of each sample. The SMRTbell Prep Kit 3.0 (PN: 102-182-700, PacBio, USA) was used for library preparation with the procedure ‘Preparing multiplexed amplicon libraries using SMRTbell prep kit 3.0’ (PN: 102-359-000, PacBio) for Primer Barcoded samples, with minor adjustments to the protocol. Finally, the 16S rRNA amplicon libraries were sequenced on the Sequel IIe PacBio System with 15 h of movie time. A negative control sample was included throughout the protocol, undergoing all steps including sampling (swabs opened at location of pig sampling but unused), DNA extraction, PCR and sequencing.

### Bioinformatics and statistical analysis

Amplicon libraries were demultiplexed using the standard SMRTlink demultiplexing procedure through *Lima* on HiFi reads with a minimum CCS Predicted Accuracy of 20 (phred score) and Barcode Score of 80. Demultiplexed reads were imported into R (version 4.2.3) (R Core Team 2023). The DADA2 package (version 1.26.0) (Callahan *et al.* 2016) was used to trim primers, filter by length (1,000–1,600 bp) and quality (*Q* > 3), dereplicate, denoise and generate amplicon sequence variants (ASVs). Samples with fewer than 1,500 filtered reads were removed. The DECIPHER R-package (version 2.26.0) (Wright 2016) was used to train a custom classifier to the species level using SILVA 138.2 (Mclaren & Callahan 2021, Quast *et al.* 2013) via LearnTaxa(). This classifier was used to assign taxonomy to the 16S sequencing data using the DECIPHER R-package function IdTaxa(). Unclassified ASVs and reads present in the negative control were removed. Classified reads were normalized by percent relative abundance at each taxonomic level. The phyloseq package (version 1.42.0) was used for relative abundance and genus-level alpha and beta diversity analyses (McMurdie & Holmes 2013) on the top 99% relatively abundant microbes. Relative abundance was examined at the phylum, genus and species levels to highlight broad and specific microbial composition patterns. Alpha and beta diversity analyses were performed at the genus level to optimize biological relevance with taxonomic resolution. This provides more meaningful insights than higher taxonomic levels, which may lack biological relevance due to result aggregation, and species level, which may lack taxonomic resolution accuracy. Beta diversity of the top 99% relatively abundant microbes was measured using Bray–Curtis dissimilarity (Sorenson 1948) and Jaccard distance (Jaccard 1908) via the ‘distance’ wrapper in the phyloseq package. Bray-Curtis is based on ASV abundance (Sorenson 1948) whereas Jaccard distance considers ASV presence or absence (Jaccard 1908). Resulting dissimilarity matrices were visualized with principal coordinates analysis (PCoA). Permutation Multivariate Analysis of Variance (PERMANOVA, 'adonis' function, 999 permutations) was used to quantify the multivariate community beta diversity between the experimental groups. The ‘betadisper’ function of the R vegan package (version 2.6–4) (Oksanen *et al.* 2022) confirmed homogeneity of group variances. Alpha diversity of the top 99% relatively abundant microbes was calculated using the abundance-based coverage estimator (ACE) metric (Chao & Lee 1992) for richness, Shannon’s Index (Shannon & Weaver 1949) for richness and evenness and Simpson’s Diversity Index (Simpson 1949) for richness and evenness by evaluating the probability that two randomly selected microbes belong to the same taxon. Measures were calculated using the ‘plot_richness’ function in phyloseq. Mann–Whitney U tests assessed univariate significance following homogeneity of variance (Levene test) and normality (Shapiro–Wilk test) tests. Benjamini–Hochberg adjusted *P*-values (False Discovery Rate; FDR) determined significant (*P* < 0.05) or trending toward significant (0.05 < *P* < 0.1) differences in vaginal microbe relative abundance.

Biomarker selection and performance analysis was completed using a modified version of a previously designed protocol (Fletcher *et al.* 2022), employing the Python library Scikit-learn (Pedregosa *et al.* 2011). Feature selection was performed using a combined approach with the supervised multivariate method Partial-Least Squares Discriminant Analysis (PLS-DA) and its associated variable importance of projection (VIP) metrics and recursive feature elimination (RFE) utilizing a decision tree classifier. Features with a VIP ≥1.5 in PLS-DA that were also selected in the RFE-CV() algorithm with the best accuracy across 5-fold cross-validation were selected as candidate biomarkers and used to train a support vector machine (SVM) classifier. A receiver operating characteristic (ROC) curve, depicting the performance of the trained SVM classifier, plotting false positive rate (100-specificity) against true positive rate (sensitivity) at various decision thresholds, was used. On the average ROC curve, the Youden’s J statistic (true positive rate - false positive rate) was calculated at each decision threshold to optimize true positive rate with false positive rate. The optimal decision threshold maximized Youden’s J statistic. Area under the curve (AUC) of the ROC curve and confusion matrix-based measures at the best decision threshold were used to evaluate the performance of the candidate biomarkers and trained SVM classifier.

## Results

Out of the 77 sows sampled, 75 (HRP: *n* = 52, INF: *n* = 23) samples were included in the final analysis after quality control of sequencing results. A total of 686,906 high-quality reads were obtained from PacBio 16S sequencing of the vaginal microbiome samples, an average of approximately 9,159 reads per vaginal microbiome sample. The negative control sample had 1,001 filtered reads, with 99.5% of these reads being classified to the *Pseudomonas* genus, 0.24% being classified to the *Bacillus* genus and 0.24% being classified to the *Phyllobacterium* genus.

### Relative abundance analysis

**Figure 1:**
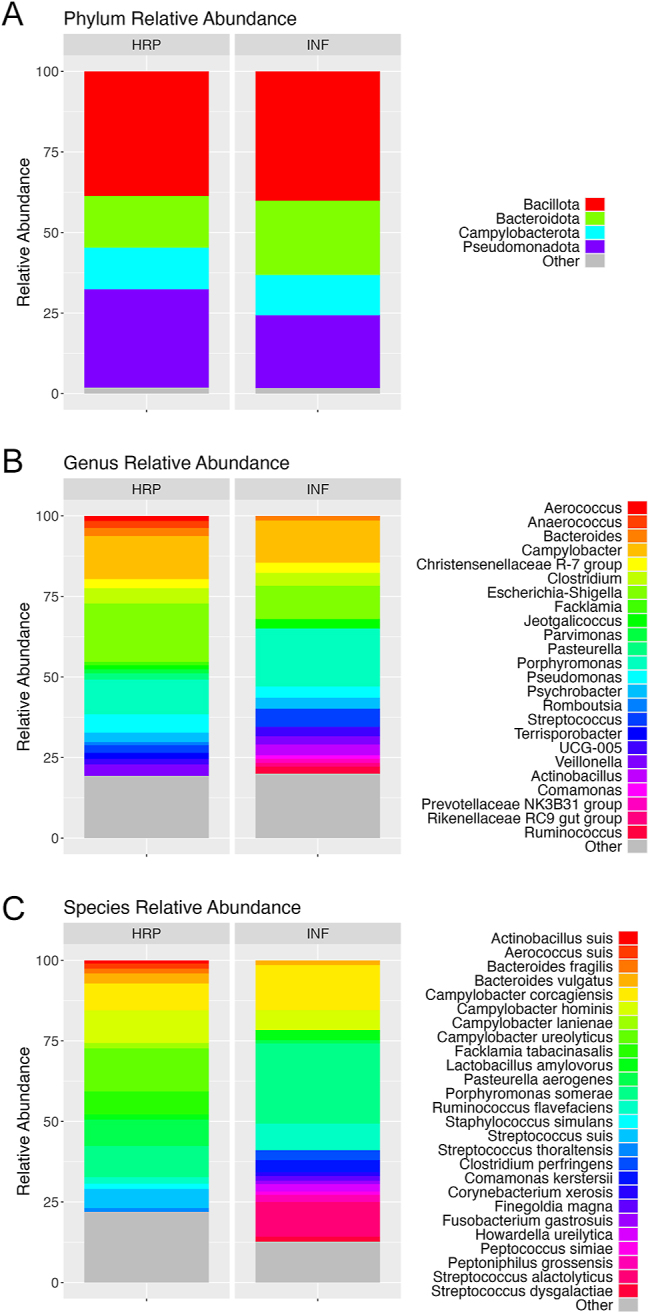
Relative abundance of vaginal microbes in HRP and INF sows at the phylum (A), genus (B) and species (C) levels. Overall, shifts in abundance of the microbes composing the vaginal microbiota communities were apparent between HRP and INF sows. Microbes with a relative abundance of ≥1% are included; the rest (relative abundance <1%) are compiled together in ‘Other’.

At the phylum level (Fig. 1A), Bacillota (41.7% in HRP, 41.2% in INF) was the most abundant in both HRP and INF microbiota. Pseudomonadota (26.4% in HRP, 19.9% in INF) was the second most abundant phylum in HRP microbiota, whereas Bacteroidota (16.7% in HRP, 24.0% in INF) was the second most abundant phylum in INF microbiota. Campylobacterota (13.2% in HRP, 13.1% in INF) was the third most abundant phylum in both HRP and INF microbiota. However, none of the differences in phylum-level relative abundance were significant between fertility groups (*P* > 0.1).

At the genus level (Fig. 1B), the most abundant genera in HRP microbiota were *Escherichia-Shigella* (18.8%), *Campylobacter* (13.7%), *Porphyromonas* (11.2%), *Clostridium* (5.4%) and *Veillonella* (3.8%). In INF microbiota, *Porphyromonas* (18.8%) was the most abundant genus, followed by *Campylobacter* (13.8%), *Escherichia-Shigella* (10.7%), *Streptococcus* (5.7%) and *Clostridium* (4.1%). Interestingly, several genera were present at a relative abundance of 1% or greater in only one group: *Aerococcus*, *Anaerococcus*, *Facklamia*, *Parvimonas*, *Pasteurella*, *Romboustia* and *Terrisporobacter* in HRP and *Actinobacillus*, *Comamonas*, *Prevotellaceae NK3B31 group*, *Rikenellaceae RC9 gut group* and *Ruminococcus* in INF. In addition, *Aerococcus and Staphylococcus* were significantly (*P* < 0.05) increased in HRP vaginal microbiota compared to INF (Table 1). In contrast, *Lachnospiraceae XPB1014 group* was significantly increased (*P* < 0.05) and *Anaeroplasma* showed a trend toward a significant increase (0.05 < *P* < 0.1) in INF vaginal microbiota compared to HRP (Table 1).

**Table 1:** Taxa with significantly (*P* < 0.05) and trending toward significantly (0.05 < *P* < 0.1) different relative abundance between HRP and INF sow vaginal microbiota communities. Bold *P* values indicate significantly different relative abundance between HRP and INF vaginal microbiota communities.

Taxonomic level/name	Corrected *P* value	Group it is increased	Mean relative abundance %	
			HRP	INF
Genus				
*Lachnospiraceae XPB1014* group	**0.005**	INF	0.19 ± 0.51	1.01 ± 1.62
*Aerococcus*	**0.024**	HRP	1.86 ± 2.83	0.18 ± 0.25
*Staphylococcus*	**0.026**	HRP	1.02 ± 1.68	0.10 ± 0.15
*Anaeroplasma*	0.084	INF	0.00 ± 0.00	0.07 ± 0.16
Species				
*Campylobacter ureolyticus*	**0.009**	HRP	13.39 ± 24.73	0.00 ± 0.00
*Staphylococcus simulans*	**0.009**	HRP	1.67 ± 3.20	0.04 ± 0.16
*Corynebacterium urealyticum*	**0.014**	INF	0.01 ± 0.08	0.28 ± 0.80
*Ruminococcus flavefaciens*	**0.014**	INF	2.01 ± 8.14	8.32 ± 18.97
*Streptococcus alactolyticus*	**0.014**	INF	0.65 ± 1.28	10.84 ± 18.05
*Streptococcus thoraltensis*	**0.017**	HRP	1.19 ± 2.79	0.00 ± 0.00
*Streptococcus suis*	**0.018**	HRP	5.95 ± 11.88	0.54 ± 1.39
*Facklamia tabacinasalis*	**0.026**	HRP	7.16 ± 12.70	0.77 ± 1.75
*Peptoniphilus harei*	**0.026**	HRP	0.55 ± 1.08	0.01 ± 0.04

At the species level (Fig. 1C), the most abundant species in HRP microbiota were *Campylobacter ureolyticus* (13.4%), *Campylobacter hominis* (10.2%), *Porphyromonas somerae* (9.7%), *Campylobacter corcagiensis* (8.4%) and *Pasturella aerogenes* (8.2%). In INF microbiota, *Porphyromonas somerae* (24.9%), *Campylobacter corcagiensis* (14.0%), *Streptococcus alactolyticus* (10.8%), *Ruminococcus flavefaciens* (8.3%) and *Campylobacter hominis* (6.2%) were the most prevalent. Interestingly, several species were present at a relative abundance of 1% or greater in only one group: *Actinobacillus suis*, *Aerococcus suis*, *Bacteroides fragilis*, *Campylobacter lanienae*, *Campylobacter lanienae*, *Facklamia tabacinasalis*, *Staphylococcus simulans* and *Streptococcus suis* in HRP and *Streptococcus thoraltensis*, *Clostridium perfringens*, *Comamonas kerstersii*, *Corynebacterium xerosis*, *Finegoldia magna*, *Fusobacterium gastrosuis*, *Howardella ureilytica*, *Peptococcus simiae*, *Peptoniphilus grossensis*, *Streptococcus alactolyticus* and *Streptococcus dysgalactiae* in INF. Moreover, *Campylobacter ureolyticus, Staphylococcus* *simulans, Streptococcus thoraltensis*, *Streptococcus suis*,* Facklamia tabacinasalis* and *Peptoniphilus harei* were all significantly increased (*P* < 0.05) in HRP vaginal microbiota. *Corynebacterium  urealyticum, Ruminococcus flavefaciens *and* Streptococcus alactolyticus* were significantly increased (*P* < 0.05) in INF vaginal microbiota (Table 1).

### Genus-level beta and alpha diversity analysis

**Figure 2:**
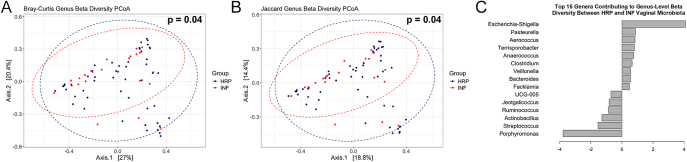
Principal coordinate analysis (PCoA) of HRP and INF sow vaginal microbiota beta diversity at the genus level based on Bray-Curtis (A) and Jaccard (B) distance metrics. HRP and INF sows displayed significant (*P* < 0.05) beta diversity divergence at the genus level. The top 15 taxa contributing to the genus community differences were also explored (C).

**Figure 3:**
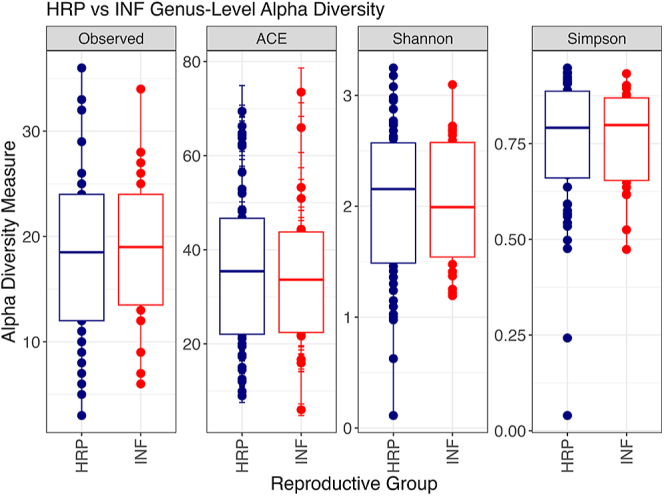
No significant alpha diversity differences were found between HRP and INF sow vaginal microbiota at the genus level. HRP and INF groups exhibited similar number of unique taxa observed and alpha diversity measures (ACE, Shannon and Simpson) at the genus level (*P* > 0.1).

The sow vaginal microbiota exhibits distinct diversity based on reproductive potential. Genus-level Bray-Curtis (PERMANOVA, *F* = 2.130, *R*^2^ = 0.028, *P* = 0.042) and Jaccard (PERMANOVA, *F* = 1.747, *R*^2^ = 0.0234, *P* = 0.041) beta diversity measures were significantly different between the vaginal microbiota of HRP and INF sows (Fig. 2A and B). When examining the top 15 *taxa* that contributed most to the PERMANOVA covariates (Fig. 2C), *Escherichia-Shigella*, *Porphyromonas*, *Pasteurella* and *Streptococcus* were the top contributing genera. In addition, the number of unique taxa observed and alpha diversity (ACE index, Shannon and Simpson) were examined at the genus level, with no significant differences (*P* > 0.10) based on reproductive potential (Fig. 3).

### Genus-level biomarker selection and model performance

**Figure 4:**
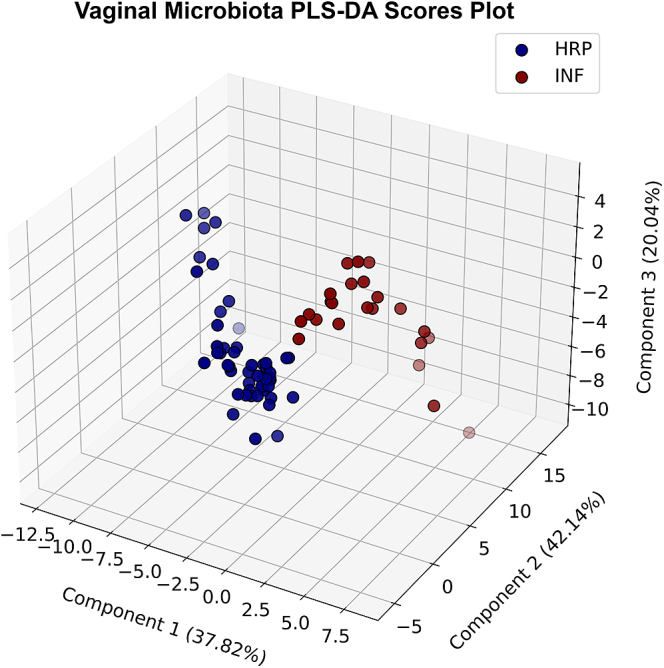
Genus-level feature selection using PLS-DA highlights distinct differences in HRP and INF vaginal microbiota composition. PLS-DA of genus-level taxonomic data displays differences in vaginal microbiota composition through a three-component PLS-DA, achieving moderate accuracy (73.3% ± 4.2), F1 Score (75.4% ± 2.1), precision (80.5% ± 2.4) and recall (73.3% ± 4.2) across 5-fold cross-validation. The percentage of explained variance for components 1, 2 and 3 was 37.82, 42.14 and 20.04%, respectively. This reflects the proportion of variance in the genus-level vaginal microbiota features captured to separate the HRP and INF groups.

**Figure 5:**
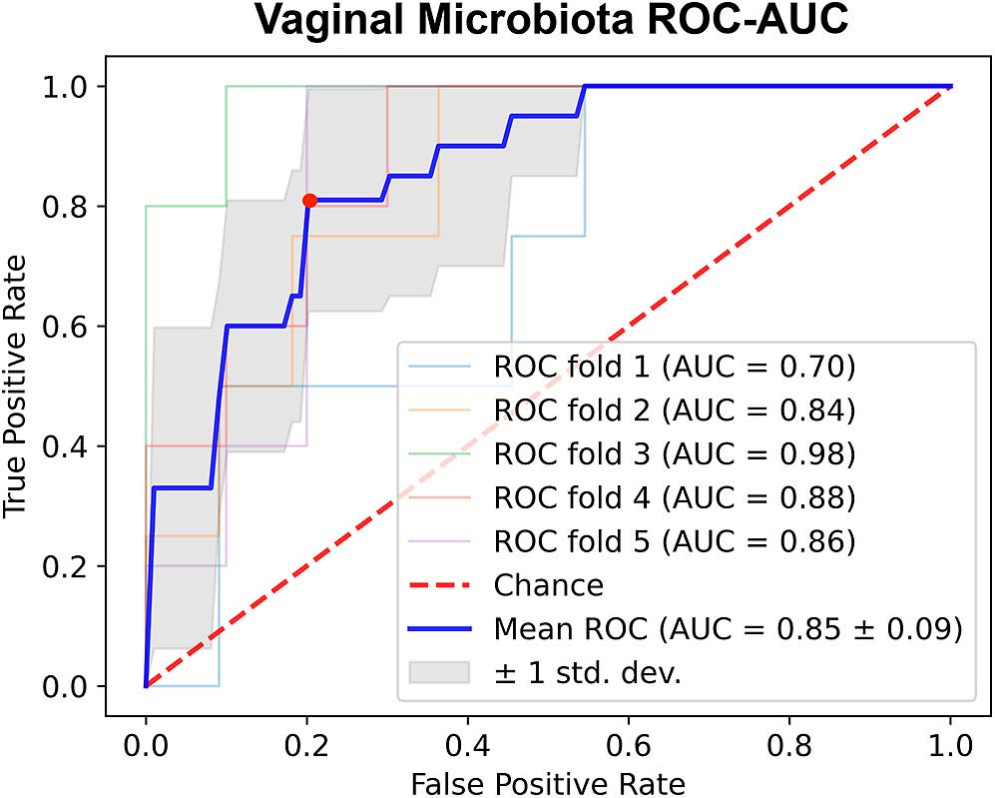
Performance of the SVM genus-level vaginal microbiota model across various decision thresholds, visualized by a ROC curve and corresponding AUC. The vaginal microbiota SVM model, trained on 42 candidate genus-level vaginal microbiota biomarkers, appeared diagnostic (AUC = 0.85 ± 0.09). The best decision threshold, determined by maximizing true positive rate and minimizing false positive rate, was 0.40, indicated by the red dot on the average ROC curve. The red line depicts a classifier with no predictive ability (AUC = 0.5); the blue line depicts the average predictive ability of the classifier over 5-fold stratified cross-validation.

PLS-DA distinguished between HRP and INF sows (Fig. 4) with moderate accuracy (73.3% ± 4.2), F1 score (75.4% ± 2.1), precision (80.5% ± 2.4) and recall (73.3% ± 4.2) across 5-fold cross-validation. The three PLS-DA components explained 37.8, 42.1 and 20.0% of the variance in genus-level vaginal microbiota features that contributed to distinguishing HRP and INF sows, respectively. The RFE model performed best when permitted to select 401 features, with 65.8% ± 15.4 accuracy. Combining these two complementary approaches using our feature selection protocol, 42 genera were selected to test as candidate vaginal microbiota biomarkers of fertility (Table 2). These candidate genus-level biomarkers performed well in the trained SVM classifier, achieving an overall AUC of 85.0% ± 9.0 across the five cross-validations in the ROC curve (Fig. 5). The best decision threshold that optimized true positive rate and false positive rate was 0.40. At this best decision threshold, the model achieved an overall accuracy of 93.3%, correctly classifying 94.2% of HRP sows and 91.3% of INF sows into their correct fertility groups.

**Table 2:** List of 42 candidate genus-level vaginal microbiota biomarkers selected via PLS-DA and RFE analysis used for training the SVM algorithm to predict sow reproductive potential.

Candidate genus-level vaginal microbiota biomarkers
*Lachnospiraceae XPB1014 group*
*Turicibacter*
*Proteiniclasticum*
*Corynebacterium*
*Terrisporobacter*
*Brumimicrobium*
*Lacticaseibacillus*
*Kocuria*
*Porphyromonas*
*Romboutsia*
*Salinicoccus*
*Sphingobacterium*
*Flaviflexus*
*Negativibacillus*
*Deinococcus*
*Staphylococcus*
*Weissella*
*Streptococcus*
*Anaeroplasma*
*dgA-11 gut group*
*Lactococcus*
*Facklamia*
*Enteractinococcus*
*Glaesserella*
*Treponema*
*Oscillospira*
*Kurthia*
*Amylolactobacillus*
*Aerosphaera*
*Tessaracoccus*
*Clostridium*
*Prevotella*
*Lapidilactobacillus*
*Aerococcus*
*Proteus*
*Quinella*
*Bifidobacterium*
*Cloacibacillus*
*Moraxella*
*Howardella*
*Yaniella*
*Ornithobacterium*

## Discussion

### HRP and INF sows exhibit distinct vaginal microbiota composition, with INF communities shifting toward anaerobic and potentially harmful bacteria

The relative abundance of the top phyla observed in our pig vaginal microbiota samples appears to mirror past investigations of the pig vaginal microbiota. Typically, pig vaginal microbiota contain Pseudomonadota, Bacillota and Bacteroidota as the top three abundant phyla (Wang *et al.* 2017, Sanglard *et al.* 2020, Zhang *et al.* 2021*a*, Kiefer *et al.* 2021, Luque *et al.* 2021, Poor *et al.* 2022). When comparing the distribution of these phyla, HRP and INF sows had a similar relative abundance of Bacillota, but INF vaginal microbiota appeared to have decreased relative abundance of Pseudomonadota and increased Bacteroidota in comparison to HRP vaginal microbiota, although these shifts were not significant. Interestingly, decreased Pseudomonadota (Poor *et al.* 2022) in the vaginal microbiota of reproductively unhealthy pigs has been previously reported. In addition, in human cases of vaginosis, an increase in Bacteroidota has been noted (Wang *et al.* 2020). It appears that, at higher taxonomic levels, HRP and INF sow vaginal microbiota exhibit similar composition and distribution. Thus, the significant differences in composition observed may be driven by variations in the abundance of specific genus- or species-level features.

Typically, the vaginal microbiome is a relatively anaerobic environment, acquiring its oxygen, glucose and nutrients from the submucosal tissues (Linhares *et al.* 2011). Due to this environment, lactic acid is a major by-product of the anaerobic metabolism of many colonizing microbes using glycogen as an energy source (Linhares *et al.* 2011). However, during vaginal microbiome dysbiosis, the environment becomes increasingly anaerobic, with reduced lactic acid and glycogen availability (Mirmonsef *et al.* 2012, Tachedjian *et al.* 2017). This is associated with colonization by a mix of opportunistic anaerobic bacteria (Marrazzo 2011, Tachedjian *et al.* 2017) that produce mixed short-chain fatty acid (SCFA) profiles (Mirmonsef *et al.* 2011, Mirmonsef *et al.* 2012, Aldunate *et al.* 2015). These SCFAs are thought to contribute to vaginal microbiome dysbiosis (Amabebe & Anumba 2020) as they have pro-inflammatory effects in the vagina (Marrazzo 2011, Mirmonsef *et al.* 2011).

Consistent with this notion, the strict obligate anaerobic genera *Anaeroplasma and Lachnospiraceae XPB1014 group* appeared elevated in INF sow vaginal microbiota, with *Anaeroplasma* trending toward significance and *Lachnospiraceae XPB1014 group* exhibiting a significant increase. These genera could play a role in fostering a dysbiotic vaginal microbiome environment, as *Anaeroplasma* has been positively correlated with SCFA concentration (Wang *et al.* 2024) and *Lachnospiraceae* is known for SCFA production in the gut microbiome (Fusco *et al.* 2023), but it remains unknown if these roles transfer to the vaginal environment. In addition, the *Lachnospiraceae* genus has been found at higher abundance in low reproductive performance sows based on number of stillborn piglets (Sanglard *et al.* 2020). In HRP vaginal microbiota, *Staphylococcus *and* Aerococcus* were significantly increased in comparison to INF vaginal microbiota. These facultatively anaerobic genera have the ability to function in a healthy aerobic vaginal environment; however, information regarding their SCFA production in the vaginal microbiome is absent. Previous research has reported higher relative abundance of the *Aerococcus* genus in the vaginal microbiota of sows with healthy vaginal discharge compared to those with unhealthy purulent discharge (Poor *et al.* 2022). The *Staphylococcus* genus also appears to be a component of healthy pig vaginal flora and was found to be more abundant in sows of HRP based on number of stillborn and mummified piglets (Sanglard *et al.* 2020). It is possible that the INF vaginal microbiota may be shifting to include harmful bacteria that function in a strict anaerobic environment and produce SCFAs that play negative roles in the health of the vaginal environment.

Some striking shifts in relative abundance at the species level were observed between HRP and INF vaginal communities. A significantly higher abundance of *Streptococcus alactolyticus* and *Ruminococcus flavefaciens* in INF vaginal microbiota was observed at the species level. *Streptococcus alactolyticus* has been isolated from the vaginal microbiota of sows (Moreno *et al.* 2016, Renzhammer *et al.* 2020); however, details on its role in the vaginal microbiome and its impact on fertility remain unknown. In addition, an increase in the species *Ruminococcus flavefaciens* in the gut microbiome has been associated with sows that did not return to estrus (Zhang *et al.* 2021*a*); however, this relationship remains to be confirmed in the vaginal microbiota. A significantly higher abundance of *Streptococcus suis*, *Facklamia tabacinasalis* and *Campylobacter ureolyticus* was observed in HRP vaginal microbiota. Interestingly, *Streptococcus suis* is a very important and researched respiratory pathogen in the swine industry (Feng *et al.* 2014); however, it is known to be a component of a healthy pig vaginal microbiota (Murase *et al.* 2019), indicating that it may play a divergent and possibly beneficial role in the vaginal microbiome. *Facklamia* is a major lactic acid-producing genus which may enhance the competitive exclusion of harmful bacteria by maintaining a healthy pH and low concentrations of other inflammatory SCFAs (Björkroth & Koort 2011, Hoyles 2014). Although the *Facklamia tabacinasalis* species has been highlighted in the vaginal microbiota in a study focusing on pelvic organ prolapse in sows (Kiefer *et al.* 2021), its involvement in fertility and how it plays a role in the vaginal microbiome remain a question. *Campylobacter ureolyticus* is a species known to colonize the human vaginal ecosystem and enhance the growth of *Lactobacillus* species (Fernández-Edreira *et al.* 2023). *Campylobacter ureolyticus* was found to be increased in sows with a low risk of pelvic organ prolapse (Kiefer *et al.* 2021); however, research on this specific species of *Campylobacter* in the sow vaginal microbiota is limited.

Consistent with the statistically significant differences observed in relative abundance, beta diversity measures of HRP and INF sow vaginal microbiota were also statistically significant based on Bray-Curtis dissimilarity and Jaccard distance PERMANOVA analysis. In contrast, HRP and INF sow vaginal microbiota did not appear to have significantly different alpha diversity at the genus level. These results align with previous studies investigating the pig vaginal microbiota in the context of negative reproductive conditions, reporting significant composition differences (beta diversity) and limited or no differences in richness and evenness (alpha diversity) (Sanglard *et al.* 2020, Zhang *et al.* 2021*a*, Kiefer *et al.* 2021, Poor *et al.* 2022). Collectively, these findings support that the vaginal microbiota of HRP and INF sows diverge primarily in bacterial composition rather than overall taxa distribution.

Overall, the differences in vaginal microbiota composition between HRP and INF sows may influence vaginal microbiome homeostasis and contribute to reproductive condition or fertility status. However, whether the observed alterations in vaginal microbiota composition are causes or consequences of sow reproductive condition remains unclear. Furthermore, many of the highlighted genera and species are understudied in the context of the pig vaginal microbiome, leaving their specific roles to be determined. Further research is needed to clarify the roles of these microbes in female pig fertility, particularly to investigate whether they exert beneficial or harmful effects on the vaginal microbiome and in what capacity they have their effect.

### SVM classifier accurately classifies sow fertility based on genus-level vaginal microbiota composition

The combined feature selection approach using PLS-DA and RFE selected 42 candidate biomarkers that enabled the SVM model to perform with strong classification performance. The trained SVM model demonstrated strong diagnostic performance for both fertility groups, achieving an area under the ROC curve of 85% ± 9.0 and overall accuracy of 93.3% at the best decision threshold. These results indicate that the model effectively discriminated between HRP and INF sows using the relative abundance data of the 42 genus-level vaginal microbiota biomarkers provided. This reinforces the importance of these selected genera in the pig vaginal microbiota and their association with sow fertility. Based on these results, it is possible that these genus-level vaginal microbes along with machine learning techniques could be applied to estimate female pig fertility in the breeding herd to make more informed management decisions. In addition, the vaginal microbiota could have predictive qualities for estimating future reproductive performance. This poses a valuable opportunity, as current breeding herd selection methods are resulting in a slower rate of genetic progress for vital litter and reproductive performance traits (Popovac *et al.* 2012, Lukac *et al.* 2016). Accordingly, breeding herd culling rates of 42.2–53.2% have been reported in North America (Ketchem *et al.* 2018) and this appears to stem from gilts in poor reproductive condition or sows with suboptimal first or second parity outcomes due to issues such as infertility, anestrus or poor reproductive performance (Hadaš *et al.* 2015, Kraeling & Webel 2015). High culling rates raise welfare concerns by increasing the number of gilts and sows needed to maintain normal production. In addition, this disturbs the flow of gilts and sows through the breeding herd, interferes with piglet production and results in an overall decrease of productivity and economic losses. An accurate vaginal microbiota model for accurately measuring fertility or reproductive potential could dramatically reduce culling related to suboptimal reproductive performance in the breeding herd, mitigating the economic and welfare impacts of current selection methods. However, future work should investigate these biomarkers in a larger population of gilts and sows to validate industry applicability and determine if the vaginal microbiota can be utilized to predict future reproductive performance before breeding herd recruitment.

## Conclusion

The findings of this study suggest that sow vaginal microbiota composition is associated with fertility status, with INF sow microbiota exhibiting a potential dominance of anaerobic microbes with harmful tendencies that may outcompete the more aerobic and beneficial microbes found in HRP sow microbiota. Substantially more research is required to define the composition of a ‘typical’ healthy pig vaginal microbiota and to better understand how its bacterial components contribute to vaginal microbiome homeostasis. Several bacterial candidates that warrant further investigation for their roles in pig vaginal microbiome function and reproductive health have been highlighted. This study also demonstrates that the vaginal microbiota can be used to accurately classify sow fertility, with 42 candidate genus-level biomarkers highlighted. Accordingly, the composition of the vaginal microbiota could serve as a tool to inform breeding herd management decisions, including the selection of female pigs for the breeding herd, enhancing efficiency by identifying infertile or low-reproductive potential female pigs before several unsuccessful rounds of AI. However, for practical on-farm application and to confirm the true predictive value of these biomarkers, further validation in a larger independent population of sows and gilts, particularly in future reproductive performance, is essential.

## Declaration of interest

The authors declare no conflicts of interest that could be perceived as prejudicing the impartiality of the research reported.

## Funding

This work was supported by Food from Thought and the Canadian First Research Excellence Fund (grant numbers 499093 and 499199).

## Author contribution statement

JL and LF conceived the study. JL secured funding, contributed to methodology design, assisted with data analysis and reviewed the manuscript. LF led methodology design, sample collection, sample and data analysis and manuscript preparation. XZ and YS assisted with sample collection and reviewed the manuscript.

## Data availability

All relevant sequencing data included in this study is available in the Agri-environmental Research Data Repository at the University of Guelph: https://doi.org/10.5683/SP3/C6Y2LN.
